# Cytogenetics of a parthenogenetic Arctic species of *Micropsectra* (Diptera, Chironomidae)

**DOI:** 10.3897/CompCytogen.v5i4.1356

**Published:** 2011-11-09

**Authors:** David L. Porter, Jon Martin

**Affiliations:** 1 Department of Genetics, The University of Melbourne VIC 3010, Australia

**Keywords:** Chironomidae, parthenogenesis, polytene chromosomes, hybridization, chromosome recombination

## Abstract

*Micropsectra sedna* (Oliver, 1976) is a parthenogenetic midge from the Canadian Arctic. The parthenogenetic mechanism is apomictic thelytoky, with a restitutional division during oogenesis, as found in other parthenogenetic Chironomidae. It is triploid, with two similar chromosome sets, and the third is relatively dissimilar, pairing little with the diploid set. Two karyotypes were observed: a single individual with eight polytene elements in the salivary glands (3n=12), considered standard, while the majority of larvae showed only seven polytene chromosomes (3n=11). Hybrid speciation is considered likely, although chromosomal recombination following the origin of thelytoky has played some part in karyotype evolution. A single morphologically distinct larva was also found, which might be the donor of the haploid chromosome set. The apomictic restitutional system is compared to that of the other, independently derived, parthenogenetic Chironomids to assess the extent of similarity between species.

## Introduction

Parthenogenesis, in the forms of arrhenotoky, deuterotoky, or thelytoky, is a quite common phenomenon in the animal kingdom (Suomalainen 1962). Thelytoky, in which females produce exclusively female progeny in the absence of genetic fertilization, is the most widespread and most mechanistically diverse form of parthenogenesis (Hartl 1971).

Thelytoky itself is present in a wide variety of forms. The mechanism for the maintenance of thelytoky may be automictic, in which at least the first meiotic division is normal, the chromosomes pairing at prophase and forming bivalents. The zygoid phase is restored by the restitution of anaphase I or metaphase II chromosome plates, fusion of second division products or endomitosis in cleavage nuclei. Alternatively the mechanism may be apomictic, in which meiotic features may be partly or wholly absent, the one or two maturation divisions being equational. Thelytoky may be complete, it being the only manner of reproduction; or it may be cyclical, where it alternates regularly, or under the influence of environmental factors, with amphimixis or arrhenotoky. Many thelytokous species are also polyploid, allopolyploidy being more common (Gregory and Mable 2005). These allopolyploids also tend to be of hybrid origin (Bullini 1994), and while it is often assumed that polyploidy and thelytoky arose together, there is no proof of this (Gregory and Mable 2005).

The eggs of many thelytokous forms require penetration by the sperm of the same or related species before they develop, but this has not been found in previously described thelytokous Chironomids (Scholl 1956, 1960; Porter 1971).

This paper will examine the cytology of *Micropsectra sedna*, a member of the chironomid subfamily Chironominae, and compare it to other independently derived, thelytokous Chironomids of the same subfamily (Porter 1971), or the subfamily Orthocladiinae (Scholl 1956, 1960).

## Material and methods

Second and fourth instar larvae of *Micropsectra sedna* were collected from Char Lake, Resolute Bay, North West Territory (now Nunavut), Canada (74°42'N; 94°53'W), inMay and July1970, packed at 4°C and air freighted to Melbourne, Australia. The stock was then split, with some set up at 15°C and the rest kept in an environment varying from 0–10°C (ave. 6°C). Only the lower temperature colony bred successfully. It was maintained in rearing units consisting of a 25cm × 25cm × 12.5cm plastic container connected to a constant air supply. The containers were placed in wooden cages with perspex sliding doors, and the sides of the cages were predominantly fine nylon mesh to allow adequate ventilation. The larvae were fed a finely ground mixture of chicken pellets, dog cubes, soya bean flour and ‘Pro-vita’ wheat hearts, alternating with several drops of a broth culture of the bacterium *Pseudomonas aeruginosa* (Schröter, 1872) (after Wool and Kugler 1968, who used *Escherichia coli* (Escherich, 1884)).

Several adults emerged from the 15°C tank, but only one oviposited and the eggs failed to develop. Between 11 June and 23 July 1970, 48 adults emerged from the refrigerator culture and 33 oviposited. The clutches varied between 96 and 372 eggs, averaging 201, of these four showed absolutely no sign of development. Of the remaining 29 clutches 2144 out of 2655 eggs hatched (81% hatchability).

Late fourth instar larvae, at the stage just after the appearance of the anlagen of the adult eye (phase 7–8 of Wülker and Götz 1968), were used to characterize the salivary gland chromosomal banding pattern. The glands were dissected from fresh larvae and stained in 1.6% orcein in 80% lactic acid - propionic acid (1:1) (Martin et al. 2006). The cover glasses were ringed with nail varnish, and the slides stored in a deep freeze.

Photographs from fresh ‘semipermanent’ preparations were used in the construction of chromosome maps. The chromosomes have been numbered 1 to 8 and arbitrarily given left and right ends. The major divisions have been numbered consecutively throughout the karyotype and each of these subdivided into minor divisions, denoted by letters, at readily identifiable bands, trying to limit the number of bands within a minor division to less than 12. The bands within these minor divisions were numbered (although the numbers are not shown in the figures due to lack of space), so any band can be identified by the number of the major division, the letter of the minor division and the number of the band within that minor division, e.g. band 16a9 can immediately be identified as in major division 16 on chromosome 3, and band 9 in minor division a. Where segments of chromosomes were heterozygous for an inversion or a deletion, the ‘diploid’ sequence has been taken as the standard sequence. The advantages of a consecutive numbering system over a system in which each chromosome is numbered independently have been discussed by Martin (1969).

For the study of oogenesis in the early embryos, whole or partial egg clutches were treated with 2% sodium hypochlorite for 10–20 sec, permitting release of the eggs from the mucopolysaccharidesheath. The eggs were fixed for 10 min in 45% acetic acid on analbuminized slide and squashed with a siliconized cover glass, which was flicked off after freezing with liquid nitrogen. The material was then dehydrated through an ethanol series, post-fixed overnight in Kahle’s fixative with water (1 part glacial acetic acid, 6 parts formalin, 15 parts ethanol, 30 parts distilled water), dehydrated, and extracted for 2 days in 1:1 methanol-chloroform mixture. The preparations were Feulgen stained (Darlington and La Cour 1962), and then rinsed for about 30 sec. in slowly running tap water (Demalsy and Callebaut 1967), dehydrated, and mounted in DePeX.

## Results

### Polytene chromosomes

*Micropsectra sedna* is triploid, based on 3n=12. There are two karyotypes present in the Char Lake collection, one with eight polytene elements in the salivary glands, the other with seven (3n=11). The biotype with eight polytene elements is considered Standard, despite the fact that it was only found once compared with about 20 of the biotype with seven polytene elements, since it has a greater likelihood of being closer to the original karyotype (see below).

**Figure 1.**
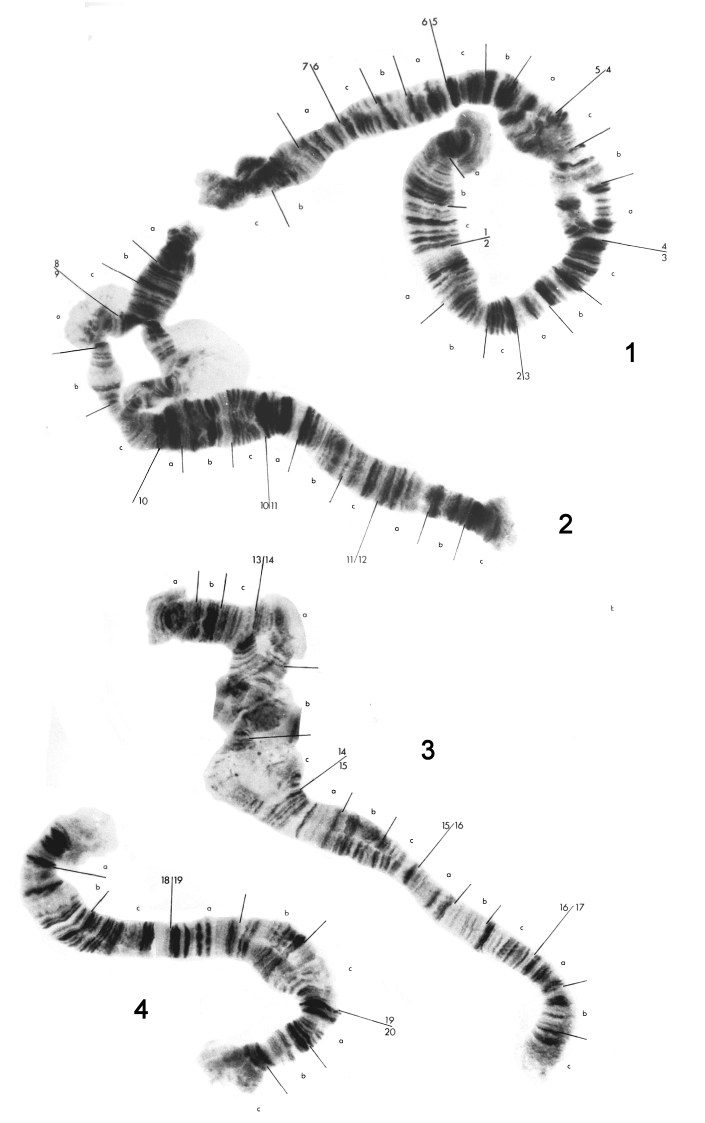
Polytene chromosome maps for chromosomes 1 to 4 of the eight-chromosome specimen of *Micropsectra sedna*.

**Figure 2.**
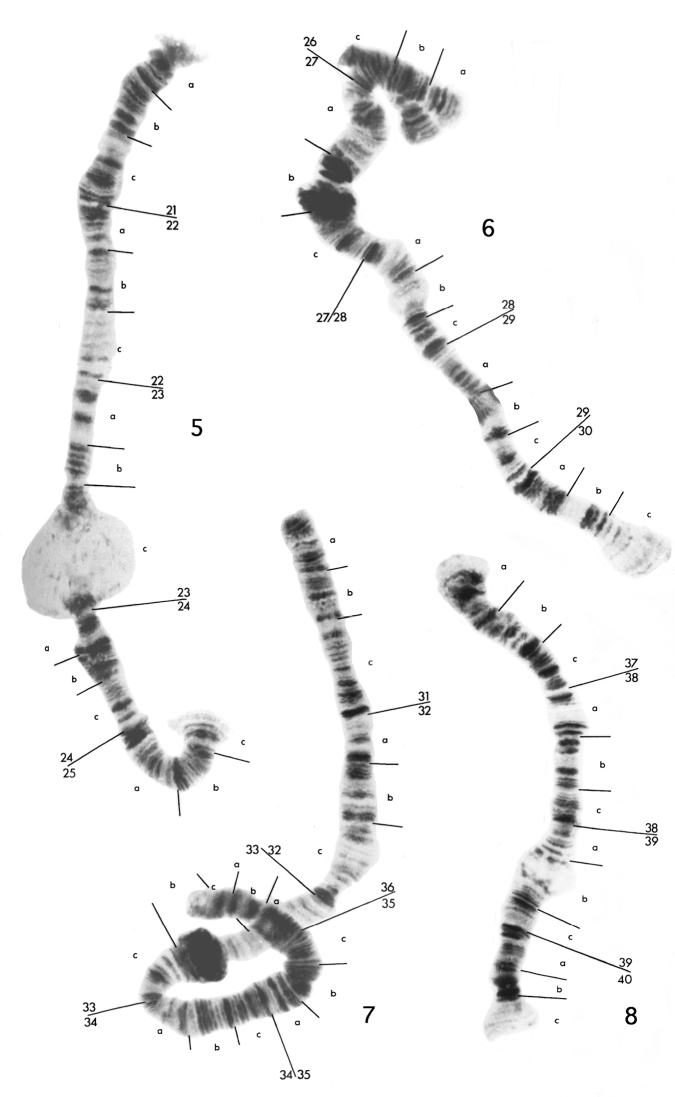
Polytene chromosome maps for haploid chromosomes 5 to 8 of the eight-chromosome specimen of *Micropsectra sedna*. The small, partially paired segment at region 26 of chromosome 6 is from region 8 of chromosome 2.

The eight chromosomes of the Standard karyotype can bedivided into two groups. Chromosomes 1 to 4 (Fig. 1) consist of two homologues, and will be referred to as the diploid set. Chromosomes 5 to 8 (Fig. 2) occur assingle entities, and will be termed the haploid set. There is some homology between members of the haploid and diploid sets, however it is impossible to trace the banding pattern of one set completely in the other, and better material would have to be used in order for this to be achieved. Breakdown of pairing is evident in the diploid chromosomes, especially in the vicinity of puffs and bulbs. These do not appear to be due to any structural rearrangements.

Chromosome 1 (Fig. 1) has been divided into seven major divisions. As with the rest of the diploid set, no centromere is obvious. There appears to be some swelling, which could be a poorly developed bulb, in 4c. There are regions of non-pairing in 4a-c, 7b-c and, in some individuals, 6a-b. Readily identifiable regions are the dark sets of bands in 2a and 2c, a dark set of at least three bands at the end of 3c and five dark regions from the middle of 5a to the end of 5c.

Chromosome 2 (Fig. 1) has been divided into five divisions, 8 to 12. There is a puff in 9a and a bulb in 9b. 9a and 9b are normally not paired and the separation may extend to the end of the chromosome at 8a. The best marker areas are a group of distinct pale separated bands in l0c followed by a dark region in 11a and the dark thick band at the start of 12c.

Chromosome 3 (Fig. 1) has been divided into five divisions, 13 to 17. Unfortunately region 14b was not distinct in any preparation There is a large swelling or bulb in 15b. The best marker areas are the dark bands at the end of 13b, the end of 15c, the end of 16a, and the start of 16c.

Chromosome 4 (Fig. 1), the smallest of the diploid set, has been divided into three divisions, 18 to 20. There are no distinctpuffs or buIbs, but the ends of this chromosome are characteristically rather diffuse. There is a tendency towards non-pairing at 19b and 19c. Obvious regions arethe dark patch in the middle of 18a, five discrete dark bands at the start of 19a, two dark bands at the start of 20a and two dark bands at the start of 20c.

Chromosome 5 (Fig. 2) has been divided into five regions,21 to 25. It is characterized by a large puff in 23c. Good markers are the dark patches of bands in 21c and 22a, pairs of bands in 22b, 22c and 23a, the dark swollen area 24b and two groups of three dark bands at the start of 25a and 25b.

Chromosome 6 (Fig. 2) has been divided into five regions, 26 to 30. It is characterized by two heterochromatic blocks in 27b, either of which could be taken for a centromere, and a small bulb at 28b. The most obvious markers are the dark region in 28c followed by a pale group of separated bands in 29a, and three dark bands at the end of 30b.

Chromosome 7 (Fig. 2) has been divided into six regions, 31 to 36. It is characterized by a heterochromatic block in 33b, which again is somewhat similar to a centromere, and a distinct constriction at the end of 32c. There are few distinct regions of this chromosome, the most obvious being the doublet at the end of 31c, and the dark patch of about four bands at the start of 35b.

Chromosome 8 (Fig. 2), the shortest of the haploid set, has been divided into four regions, 37 to 40. It has a small puff in 39b, which is quite variable in size, frequently being absent in the biotype with seven polytene elements. The best marker regions are the dark patch of close bands at the end of 37c, five dark separated bands at 38a-38b, the dark bands at the start of 40a and the two dark pairs of bands in 40b.

### Homology between the chromosome sets

It is usually rather difficult to pick homology, because generally the diploid set is not overly stretched and there are regions of non-pairing, whereas the haploid chromosomes are quite stretched.

Region 8 in chromosome 2 and region 26 in chromosome 6 appearto be the same because they have been found to pair occasionally, as shown in Fig 2. This is the only homologous pairing seen between members of the haploid and diploid sets. Also the sequence 10a3 to 11a8 in chromosome 2 appears to have been inverted and ispresentas 29c6 to 28b6 in chromosome 6.

In chromosomes 3 and 5, there is a degree of similarity between 15b17–16b2 and 21b4–22c2, likewise between 13a-c and 25c-a.There also appears to be close correspondence between chromosomes 4 and 8. The sequence from the start of 18a to somewhere in the swollen 19c is probably homologous to the sequence from the start of 37a to the puff in 39b. There are also similarities between 20a8–20b3 and 39b7–39c3, and between the dark bands at the start of20a and 40a. The end of chromosome 4 is too indistinct to postulate further homology. This leaves chromosome 7 as the possible homologue of chromosome 1, and there are some regions of possible homology, such as 5c-6b with 34a-35a, to support this suggestion.

### Chromosomal rearrangements

The biotype with seven polytene elements, which is the most common in the material analysed, differs from the biotype with eight polytene elements by the break up of chromosome 5 and its addition to chromosomes 3 and 8.

**Figure 3.**
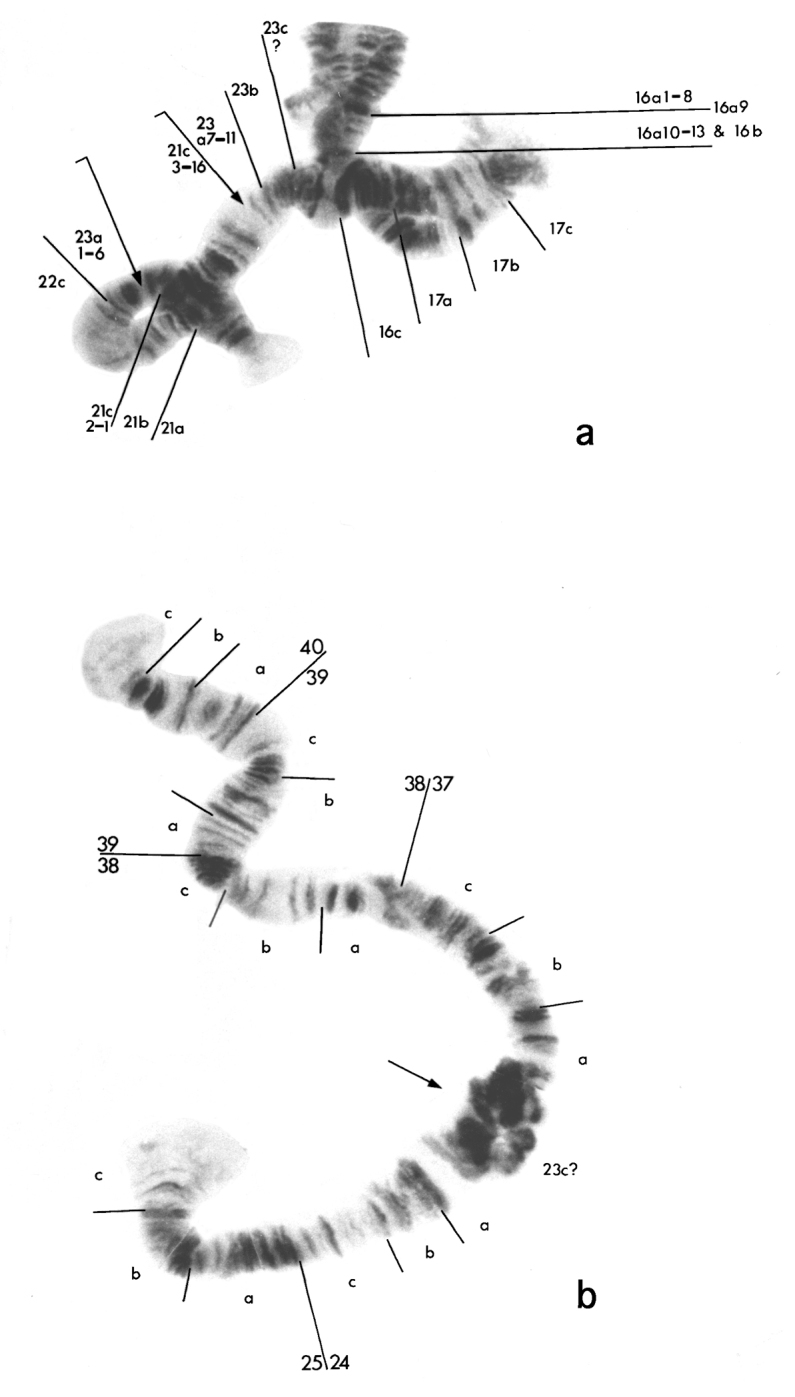
Modified chromosomes of the seven-chromosome biotype of *Micropsectra sedna*. **a** Part of chromosome 5, regions 21a to 23c7, attached to chromosome 3. Arrows show the inversion in the chromosome 5 segment. C3 – chromosome 3; C5 – chromosome 5 **b** Diagrammatic representation of chromosomes in **a** **c** Other part of chromosome 5 attached to chromosome 8. Arrow indicates the point of fusion.

There appears to have been a break in one of the homologues of chromosome 3, between the two dark groups of bands in 16a just after 16a9. A second break occurred in chromosome 5 just before the puff in 23c. In addition, chromosome 5 has an inversion of about 21c3 to 23a6 (Inv21c3–23a6), the breakpoints of which are indicated by arrows below. The region 21a to 23c, carrying the inverted sequence, is then joined to one of the chromosome 3 homologues at 16a10 (Fig. 3a) to give the following complex:

**Figure:**
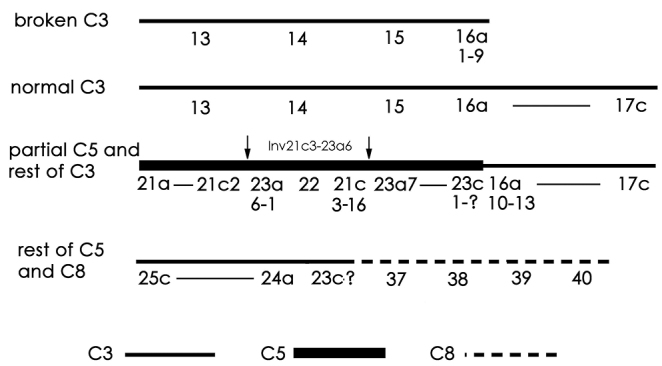


The reciprocal product is a deleted chromosome 3, comprising regions 13 to 16a9. An unresolved question is whether this deleted chromosome has a telomere at 16a9 and, if so, where it came from. The other chromosome 3 homologue remains unaltered. The rest of chromosome 5 is joined at its proximal end (23c), to region 37 at the left end of chromosome 8 (above and Fig. 3b).

### Maturation divisions

**Figure 4.**
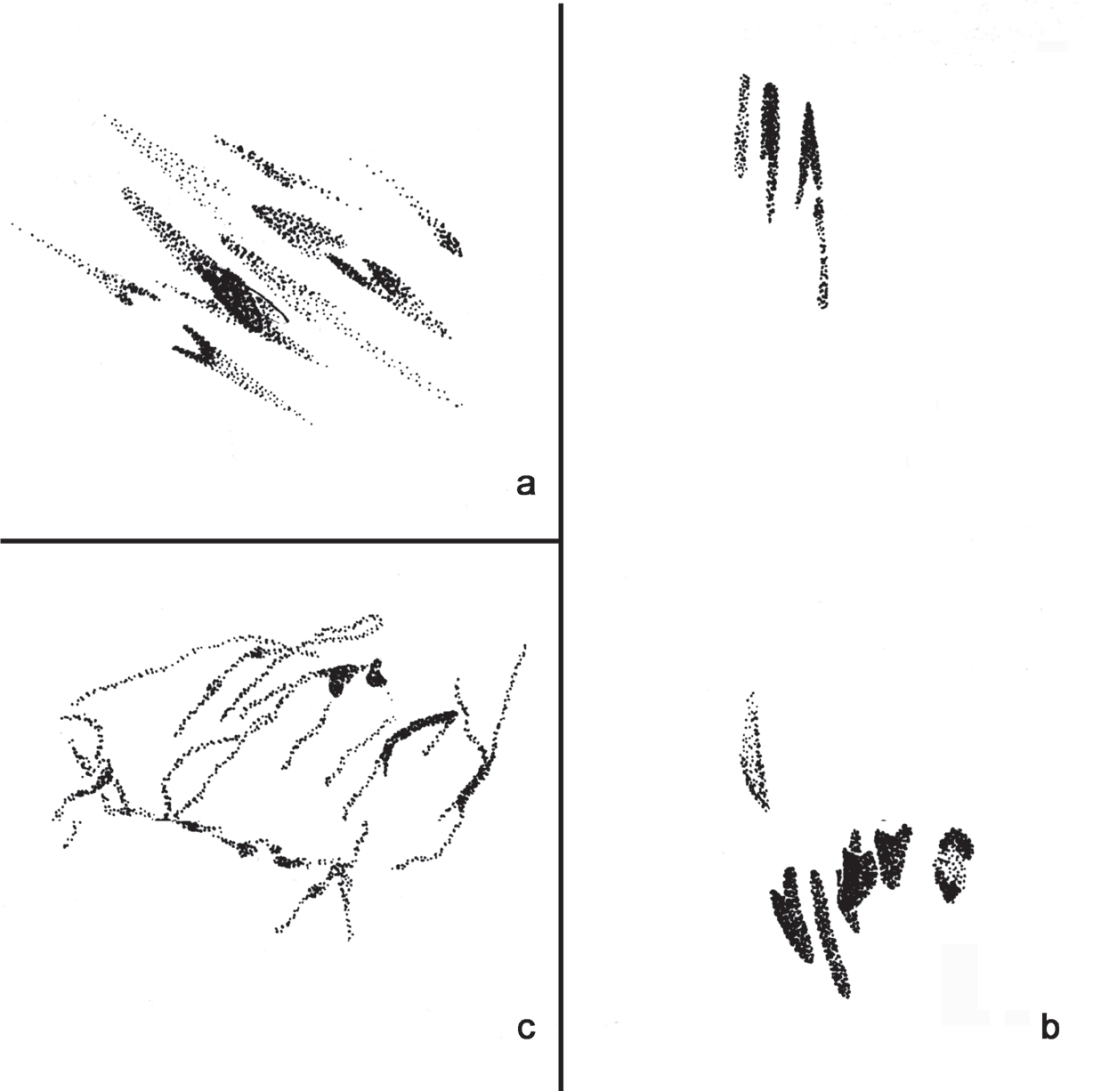
Maturation divisions: **a** Early anaphase I **b** Late anaphase I **c** “Restitutional” stage.

At early anaphase I, after 60 min, the chromosomes are pale staining and quite despirallized (Fig. 4a). By the end of anaphase, after 90 min, they are very condensed and stain strongly (Fig. 4b). At least 7 chromosomes are present at some poles although 6 or less than 6 are usually seen. After 120 min very despirallized, disorganized stages are present (Fig. 4c); these probably correspond to the restitution stage reported in *Paratanytarsus grimmi* (Schneider, 1885) (formerly *Lundstroemia parthenogenetica* Freeman, 1962) (Porter 1971). No second anaphase was seen.

Up to what is regarded as the restitution stage, the maturation mechanism appears to be essentially the same as *Paratanytarsus grimmi* (Porter 1971), differing only in the degree of chromosomal contraction, although this may itself be an artefact.

### Occurrence of a diploid form

One larva out of the 31 analysed was found to be diploid, 2n=8, rather than triploid. This diploid larva was in extremely poor condition and it could not be sexed. It also was not possible to obtain publishable photographs of the polytene chromosomes or to compare the banding pattern with those of the triploids.

There are four polytene chromosomes, showing considerable non-pairing of homologs; however there does not appear to be any inversion heterozygosity. Three chromosomes are quite long, two of them having heterochromatic blocks which may be centromeres. The small fourth chromosome has a number of puffs and is unpaired for most of its length.

The larval morphology of the diploid differed somewhat from the triploids, although still *Micropsectra*. The size and shape of the labial plates and mandibles of the two types were found to be similar, however the antennae and the length of the setae on the ultimate abdominal segment were markedly different.

## Discussion

### Origin of the diploid and haploid chromosome sets of Micropsectra sedna

As indicated above, the presumed standard polytene chromosome complement of this species comprises four diploid chromosomes and four haploid chromosomes, with relatively different banding sequences. As well, two of the haploid set have a large heterochromatic block, which is postulated to be the centromere, while there is no development of heterochromatin to indicate the centromere locations in the diploid set. Despite these differences and the very limited pairing observed between members of the two sets, there appears to be some relationship between each of the chromosomes of the haploid set and a chromosome of the diploid set: C1 with C7, C2 with C6, C3 with C5, and C4 with C8. This suggests that *Micropsectra sedna* is a triploid of hybrid origin, with a diploid chromosome set (C1-C4) from one parent, and a haploid set (C5-C8) from the other parent. The extent of difference between the two sets suggests that the two species may not have been particularly closely related. One possibility is that the single diploid larva in our sample contributed the haploid set of *Micropsectra sedna*, but the poor condition of the chromosomes of the specimen make this only speculation, but supported by the presence of heterochromatic blocks in two chromosomes, as seen in the haploid set of *Micropsectra sedna*. The morphological differences of the diploid larva are not of such a nature as to immediately rule out the possibility of viable hybridization. *Micropsectra* is one of the most species rich genera in the Chironomidae (Ekrem et al. 2010) with more than 130 species in the Holarctic region. However, only a few have been studied cytologically (Michailova 1989), and these are not closely related to *Micropsectra sedna*. So, while there may be potential for hydridization in a speciose genus, we cannot suggest what the parental species of *Micropsectra sedna* might be.

It is also possible that the differences between the two chromosome sets are due to extensive recombination and mutation of the chromosomes subsequent to the development of thelytoky. There are some factors of the arctic environment that have been suggested as explaining why triploid thelytoky and selection, including chromosomal recombination and mutation may be advantageous. Most of these were outlined by Downes (1962), and relate to the arctic as a marginal and variable environment for insects. Downes suggested that polyploidy, hybridization and apomixis provided stable genetic variability that permits the insects to successfully exploit this extreme environment. The adoption of thelytoky also permits a species to avoid another of the problems of this extreme environment - the limited time when temperatures are high enough to permit flight, and the strong winds that limit the ability to swarm and hence find partners. Some species overcome this problem by mating on the ground, but this still requires males, which are more susceptible to cold than are females. Oliver and Danks (1972) found that arctic species of Tanytarsini tend to have fewer males than females. Adoption of thelytoky can therefore be further advantageous in eliminating the need for these more susceptible males.

While Downes (1962) saw the absence of meiosis as leading to a stable genotype, this need not necessarily be true. Polyploidy provides genetic variability on which selection can act, perhaps to adapt the triploid to a niche with less competition from the parental forms. The high levels of chromosomal rearrangement in *Micropsectra sedna* could also have selective advantage if they have occurred subsequent to the origin of the polyploidy, even if no hybridization was involved. Chromosomal recombination can occur freely in the absence of meiosis and potential phenotypic variability could occur by bringing different genes into close proximity and, as recent genomic studies suggest, inactivating existing genes or creating new ones at the break points (Furuta et al. 2011). Clones with advantageous mutations would become more prevalent than those with less advantageous genomes. This may well be the explanation for the greater prevalence of the seven-chromosome form of *Micropsectra sedna* compared to the Standard eight-chromosome form.

### The restitutional mechanism

Where the mechanism of thelytoky is apomictic, restitution is one possible means of restoring the chromosome number. Restitution is the annulment of a maturation division by the immediate reunion of the separating elements, and although rather widespread in occurrence is still quite a rare phenomenon. It appears to be the normal mechanism in parthenogenetic Chironomids, as it has been recorded in the Orthocladiine parthenogens examined by Scholl (1956, 1960) and in the Tanytarsine *Paratanytarsus grimmi* (Porter 1971), as well as in the present species. After prophase the univalents assemble at the spindle equator, and undergo an abortive first division, followed by the formation of a restitutional spindle on which an apparently normal mitotic division takes place. The resulting nuclei each take part in segmentation.

There are certain specific differences relating to the arrangement of the chromosomes in prophase, the degree and pattern of despirallization during the abortive division, and the extent of development of the second division equatorial plate, but the mechanism is identical in all of them. Scholl (1960) indicated that orientation of the centromere region is random in the abortive first division of the Orthocladiines because the univalents separated to the poles at anaphase in quite a variablemanner, while Porter (1971) found that variation in *Paratanytarsus grimmi* was rare, perhaps 1 in 20. Scholl observed some terminal associations during prophase of the Orthocladiines, which were not seen in *Paratanytarsus grimmi*, and it is possible that these persist to metaphase and have some effect on the orientation of the univalents at metaphase I. There were insufficient anaphases to make any comment about *Micropsectra sedna* in this regard.

Since thelytoky in these diverse Chironomids must have arisen independently, why have they all followed the same mechanism? It might be suggested that the ability to develop without the incorporation of the male genome is a rather common occurrence, but perhaps latent and not manifested until triggered by hybridization, environmental stimulus or mutation. Whatever the stimulus, the maturation will then proceed in a genetically determined manner. An important component of this genetic determination may be the ability of the centrosome to self-reassemble in the divisions of parthenogenetic insects (Yang et al. 2008). Beyond this, it may also be logical to assume that the genetic resemblance that places organisms in the same taxonomic grouping, may lead to a similar mechanism of thelytoky being more likely to become established - in this case, apomixis with a restitutional division.

This does not mean that the mechanisms will be identical in all details, as noted above. The significance of the differences in the extent of despirallization during the restitution division is unknown. Scholl’s (1960) figures indicate that the chromosomes of *Limnophyes virgo* Remmert, 1953, more so than the other species he studied, undergo considerable extension during anaphase I, particularly near the centromere region. *Micropsectra sedna* also shows this peculiarity, far more so than *Paratanytarsus grimmi* (Fig. 4, c.f. Fig. 2, Porter 1971). However, in this case it is possible that the despiralization observed in *Micropsectra sedna* may be an artefact of the laboratory rearing conditions. As an arctic species it would rarely experience temperatures above about 2–3°C in nature, whereas the eggs were kept at about 5–6°C in the laboratory and the despiralization may have been a consequence of this.

The parthenogenetic system of *Micropsectra sedna* therefore more closely resembles that of the other known Tanytarsine parthenogen *Paratanytarsus grimmi*, in being triploid, with chromosomal polymorphism of probable hybrid origin. The parthenogenetic species in the relatively distant Tribe Orthocladiinae, studied by Scholl (1956, 1960), are more variable in that only some are triploid, some have additional germ-line limited (K-) chromosomes, and extent of polymorphism varies from a single heterozygous inversion to complex intrachromosomal rearrangement. *Micropsectra sedna* does seem to share chromosomal despirallization during the restitution division with *Limnophyes virgo*, more so than with the more closely related *Paratanytarsus grimmi*, but this feature seems to be a sporadic species-specific character.
